# Visceral fat area as a prognostic biomarker in gastric cancer: a systematic review and meta-analysis of survival outcomes

**DOI:** 10.3389/fonc.2025.1532200

**Published:** 2025-09-08

**Authors:** Lei Liu, Shufu Hou, Bing Yan, Ruiqi Hao, Linchuan Li, Dandan Song

**Affiliations:** ^1^ Department of Neurology, Shandong Provincial Third Hospital, Cheeloo College of Medicine, Shandong University, Jinan, China; ^2^ Department of Gastrointestinal Surgery, Central Hospital Affiliated to Shandong First Medical University, Jinan, China; ^3^ Department of Gastrointestinal Surgery, Xintai City People’s Hospital, Xintai, Shandong, China; ^4^ Department of General Surgery, The First Affiliated Hospital of Shandong First Medical University, Jinan, China

**Keywords:** gastric cancer, visceral fat area, overall survival, disease-free survival, meta -analysis

## Abstract

**Background:**

Visceral fat area (VFA) has been suggested as an alternative to body mass index (BMI) for evaluating the effects of obesity and fat distribution. Although VFA has shown potential as a predictor of perioperative complications and surgical duration, its prognostic value in gastric cancer patients remains contentious. This meta-analysis aimed to elucidate the prognostic significance of VFA in this patient population.

**Methods:**

We searched the PubMed, Cochrane Library, CNKI, and EMBASE databases, including literature published up to July 31, 2024, to investigate the prognostic implications of VFA in postoperative and non-surgical gastric cancer patients. Outcome measures encompassed overall survival (OS), relapse-free survival (RFS), disease free survival (DFS).

**Results:**

A total of 6,054 eligible patients were selected from 12 studies. The results indicated a significant association between elevated VFA and improved OS and DFS/RFS (OS: HR 0.76, 95% CI 0.65-0.90, p = 0.001; DFS: HR 0.84, 95% CI 0.75-0.95, p = 0.004). Subgroup analyses were conducted to assess the robustness of these findings.

**Conclusions:**

This meta-analysis demonstrated that among patients with gastric cancer who underwent surgery or other treatments, elevated VFA was significantly associated with improved OS and DFS/RFS. Consequently, VFA may serve as a useful prognostic indicator for assessing the prognosis of gastric cancer patients following treatment. However, further prospective studies are necessary to validate these findings and confirm the reliability of VFA as a prognostic marker.

**Systematic review registration:**

https://inplasy.com/, identifier INPLASY202490078.

## 1 Introduction

Gastric cancer is a prevalent malignancy, accounting for 6.8% of cancer-related mortality worldwide (1). It is the fifth most commonly diagnosed cancer globally and ranks third in cancer-associated deaths (2). Gastric cancer is characterized by its asymptomatic onset, rapid progression, high degree of malignancy, and poor prognosis (3–5). The majority of patients are diagnosed at an advanced stage, significantly limiting the benefits of surgical intervention; many may even lose the opportunity for curative resection (6, 7). This highlights the necessity for more sophisticated and comprehensive therapeutic strategies aimed at disease control, symptom palliation, and improving overall survival and quality of life. While multimodal treatments such as combination chemotherapy (8) and immunotherapy (9) have demonstrated some efficacy in extending survival and enhancing quality of life, their overall clinical impact remains suboptimal.

Notably, with economic development, advancements in healthcare infrastructure, increased public health awareness, and the widespread use of antibiotics, the incidence of gastric cancer has declined compared to historical trends (1). However, an alarming rise in incidence is being observed in younger individuals, particularly those with obesity (10). Multiple studies have identified obesity as a significant risk factor not only for cardiovascular diseases but also for various malignancies, negatively influencing oncological outcomes (11, 12). Specifically, visceral obesity may facilitate tumorigenesis through the induction of adipose tissue inflammation and systemic inflammatory responses, leading to dysregulation of key signaling pathways in the adipose microenvironment and the tumor microenvironment (TME) (13, 14). Body mass index (BMI), defined as the ratio of body weight (kg) to the square of height (m²), is a widely utilized metric for evaluating excess adiposity and its severity (15, 16). While BMI is advantageous due to its simplicity, cost-effectiveness, and reproducibility, it fails to consistently reflect body fat distribution across individuals. Significant variability exists in body fat percentage at the same BMI across different age groups, genders, and ethnicities (17–19). In contrast, VFA is a more precise indicator of adipose tissue dysfunction and has been strongly linked to a range of obesity-related comorbidities (20, 21). Additionally, VFA has been proposed as a superior metric to BMI in assessing perioperative risk (22). Several studies have reported that increased VFA is associated with higher rates of postoperative complications in gastric cancer surgery, such as pancreatic fistula, anastomotic leakage (23), and surgical site infections (24). Moreover, elevated VFA has been correlated with worse clinical outcomes in patients with breast cancer, pancreatic cancer, and hepatocellular carcinoma (25–27).

Despite these findings, the prognostic significance of VFA in gastric cancer patients undergoing surgical or non-surgical treatment remains a matter of debate. Thus, elucidating the impact of visceral fat area on the prognosis of gastric cancer patients is of paramount importance. Quantitative measurement of body composition, including muscle and fat distribution, through computed tomography (CT) has been established as a reliable method for evaluating body composition (28–31), and has become a burgeoning area of research in oncology. Clinically, patients typically undergo axial abdominal CT scans in the supine position, with measurements commonly taken at the levels of L3, L4, and the umbilicus. Image analysis software is then employed to delineate visceral and subcutaneous fat compartments. The density of adipose tissue on CT images generally ranges between -150 and -50 Hounsfield units (HU), allowing for accurate identification and quantification of fat areas (measured in cm²).

This study employs a systematic review and meta-analysis to evaluate the association between VFA and the prognosis of gastric cancer patients, providing clinicians with a novel prognostic tool for use in clinical decision-making.

## 2 Materials and methods

### 2.1 Search strategy

This systematic review and meta-analysis were conducted in accordance with the guidelines outlined in the Preferred Reporting Items for Systematic Reviews and Meta-Analyses (PRISMA) (32). Two independent researchers systematically searched PubMed, Embase, CNKI, and the Cochrane Library to identify studies related to the prognostic significance of VFA in both postoperative and non-surgical gastric cancer patients. The search encompassed relevant studies from the inception of these databases until July 31, 2024. We used the following keywords to investigate the predictive significance of VFA in gastric cancer patients: “Gastric Cancer” or “Stomach Neoplasms” or “Gastric Neoplasms” or “Gastric Adenocarcinomas” or “Stomach Cancer” or “Stomach carcinoma” or “Gastric malignancy,” and “Visceral Fat” or “Abdominal Fat” or “Intra-Abdominal Fat” or “Adipose Tissue” or “Visceral fat area” or “Intra-abdominal fat area” or “Visceral adiposity area.” In addition to using free-text terms and Medical Subject Headings (MeSH) for searching within titles and abstracts, we screened the references of selected articles to ensure a comprehensive search.

### 2.2 Inclusion and exclusion criteria

#### 2.2.1 Inclusion criteria

(1) Patients were diagnosed with gastric cancer through a comprehensive evaluation, including imaging studies, serum tumor marker tests, and histopathological biopsy; (2) Visceral fat area (VFA) was calculated using the L3-CT imaging level; (3) Studies provided long-term survival data such as overall survival, relapse-free survival, or disease-free survival; (4) Hazard ratios (HR) and 95% confidence intervals (CI) could be obtained either directly from the literature or indirectly through calculation.

#### 2.2.2 Exclusion criteria

(1) reviews, case reports, case series, conference abstracts, or commentaries; (2) studies with insufficient data; (3) nonclinical or nonhuman studies; (4) studies with overlapping or duplicate data; (5) studies without preoperative CT-confirmed visceral fat area.

### 2.3 Data extraction and quality assessment

Two researchers independently extracted data from eligible studies. Any discrepancies were resolved through discussion or consultation with a third researcher. The extracted data included: first author’s name, publication year, country of study, study design, sample size, mean or median age of included patients, gender distribution, treatment modalities, and survival analyses (including hazard ratios and corresponding 95% confidence intervals for overall survival and disease-free survival). The quality of the studies was assessed using the Newcastle-Ottawa Scale (NOS), which covers three aspects: selection (0–4 points), comparability (0–2 points), and outcome assessment (0–3 points). Each of the two researchers scored the three aspects across eight questions, with a total score ranging from 0 to 9. Studies with a score above 6 were considered high-quality studies (33).

### 2.4 Data statistics

Statistical analysis was conducted using Stata SE (version 12.0; StataCorp, College Station, Texas, USA). Heterogeneity among the studies was assessed with Cochran’s Q-test and I² statistics. When heterogeneity was not significant (P ≥ 0.10 or I² < 50%), a fixed-effects model was used; otherwise, a random-effects model was applied in the presence of significant heterogeneity (P < 0.10 or I² ≥ 50%). For survival data, including OS and DFS or RFS, hazard ratios (HR) and their 95% confidence intervals (CI) were calculated. Statistical significance was defined as P < 0.05. Publication bias was evaluated by examining the symmetry of the funnel plot and using methods such as Egger’s linear regression and Begg’s rank correlation test, with a P-value < 0.05 suggesting potential publication bias. Sensitivity analysis was performed to determine the impact of individual studies on OS and DFS or RFS. To investigate the sources of heterogeneity, subgroup analyses were conducted based on treatment modalities, sample sizes, cutoff values, and analytical models.

## 3 Results

### 3.1 Study selection and characteristics

**Figure 1:**
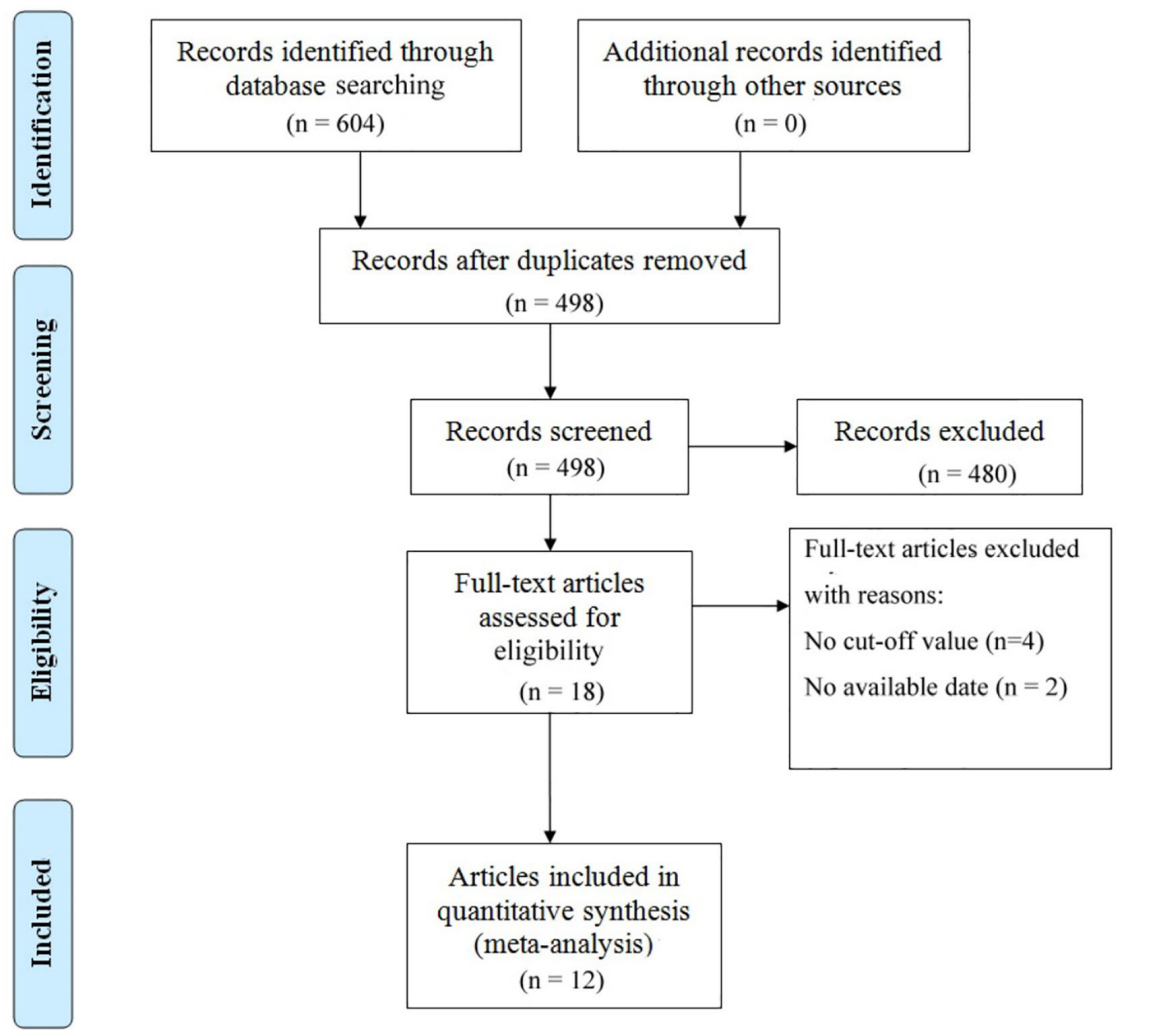
Prisma flowchart illustrating the literature selection process.

The literature selection process is comprehensively outlined in **Figure 1**. In accordance with the previously established search strategy, an initial pool of 604 articles was identified. After the removal of duplicates, 498 unique studies remained. Subsequently, a rigorous screening of titles and abstracts based on predefined inclusion and exclusion criteria resulted in the exclusion of 480 studies. An additional six studies were excluded due to the unavailability of full-text access. Ultimately, 12 studies assessing the correlation between VFA and gastric cancer prognosis were included in the final analysis (14, 34–44). **Table 1** provides a summary of the key characteristics of these studies. Published between 2014 and 2024, ten studies were conducted in China, while the remaining two originated from Japan and South Korea. Of these, eight were retrospective in design, while four were prospective. The sample sizes across the studies ranged from 110 to 1,147, with a cumulative total of 6,054 patients. Nine studies focused on surgical interventions alone, while two included combination regimens involving ICIs; one study did not comprehensively report treatment details. Disease-free survival was reported in seven studies, while overall survival (OS) was documented in 11 studies. Quality assessment using the Newcastle-Ottawa Scale (NOS) yielded scores ranging from 6 to 8 across the included studies, reflecting a generally high level of methodological rigor. The detailed NOS scores are presented in **Table 2**.

**Table 1:** Baseline characteristics of included studies.

Study, year	Country	Duration	Study design	Sample size	Age	Gender (M/F)	Follow-up (months)	Stage	Treatment	Cut-off (cm2)	Survival outcome	Analysis	NOS
Kim 2014 (37)	Korea	2005-2008	Retrospective	304	median:60(25-86)	207/97	median:62(26-97)	I-III	Surgery	150	OS	U	7
Wang 2017	China	2009-2013	Prospective	859	median:64	672/187	median:60.9	I-III	Surgery	100	OS,DFS	U,U	7
Dong 2020	China	2014-2019	Prospective	1147	median:65	848/299	median:35.2	I-III	Surgery	100	OS,DFS	U,U	7
Taniguchi 2020	Japan	2010-2015	Retrospective	567	NR	393/184	NR	I-IV	Surgery	100	RFS	M	7
Huang 2021 (44)	China	2014-2019	Retrospective	597	median:72	463/134	median:33.5	I-III	Surgery	100	OS,DFS	U,U	7
Zhang 2021 (36)	China	2016-2018	Retrospective	110	median:61.5(53-67)	80/30	median:23	I-IV	Surgery+neoadjuvant treatment	120	OS,DFS	M,M	8
Bian 2022 (34)	China	2013-2016	Retrospective	142	NR	NR	median:70	I-IV	Surgery	71.1	OS	M	8
Liu 2022 (43)	China	2011-2016	Retrospective	379	56.65 ± 10.68	254/125	NR	I-III	Surgery	87.1	OS	U	6
Wu 2022 (38)	China	2014-2018	Prospective	585	median:69	441/144	median:56.4	I-III	Surgery	M:96.1 F:105.2	OS,DFS	U,U	8
He 2023 (35)	China	2019-2022	Retrospective	158	median:63(56-69.3)	129/29	NR	IV	Dual PD-1 and HER2 blockade	M:156 F:74	OS,DFS	U,U	6
Li 2023 (14)	China	2012-2020	Prospective	299	NR	NR	median:41(0.1-296)	NR	NR	102	OS	M	8
Zhuang 2024 (42)	China	2013-2019	Retrospective	907	NR	NR	NR	I-IV	Surgery	100	OS	U	6

M, male; F, female; NR, not report; OS, overall survival; DFS, disease-free survival; U, univariate; M, multivariate; NOS, Newcastle-Ottawa Scale.

**Table 2:** Newcastle-ottawa scale (NOS) for quality assessment.

Studies	Selection				Comparability	Outcome			Scores
	A	B	C	D	E	F	G	H	
Kim 2014 (37)	★	★	★	★	★	★	★	–	7
Wang 2017	★	★	★	★	★	★	★	–	7
Dong 2020	★	★	★	★	★	★	★	–	7
Taniguchi 2020	★	★	★	★	★★	★	–	–	7
Huang 2021 (44)	★	★	★	★	★	★	★	–	7
Zhang 2021 (36)	★	★	★	★	★★	★	★	–	8
Bian 2022 (34)	★	★	★	★	★★	★	★	–	8
Liu 2022 (43)	★	★	★	★	★	★	–	–	6
Wu 2022 (38)	★	★	★	★	★★	★	★	–	8
He 2023 (35)	★	★	★	★	★	★	–	–	6
Li 2023 (14)	★	★	★	★	★★	★	★	–	8
Zhuang 2024 (42)	★	★	★	★	★	★	–	–	6

A study may receive a maximum of one star for each numbered item in the Selection and Outcome categories. A maximum of two stars may be given for Comparability, as directed by the NOS. ★It stands for one point; ★★It stands for two points.

### 3.2 Association of VFA with OS and DFS/RFS

**Figure 2:**
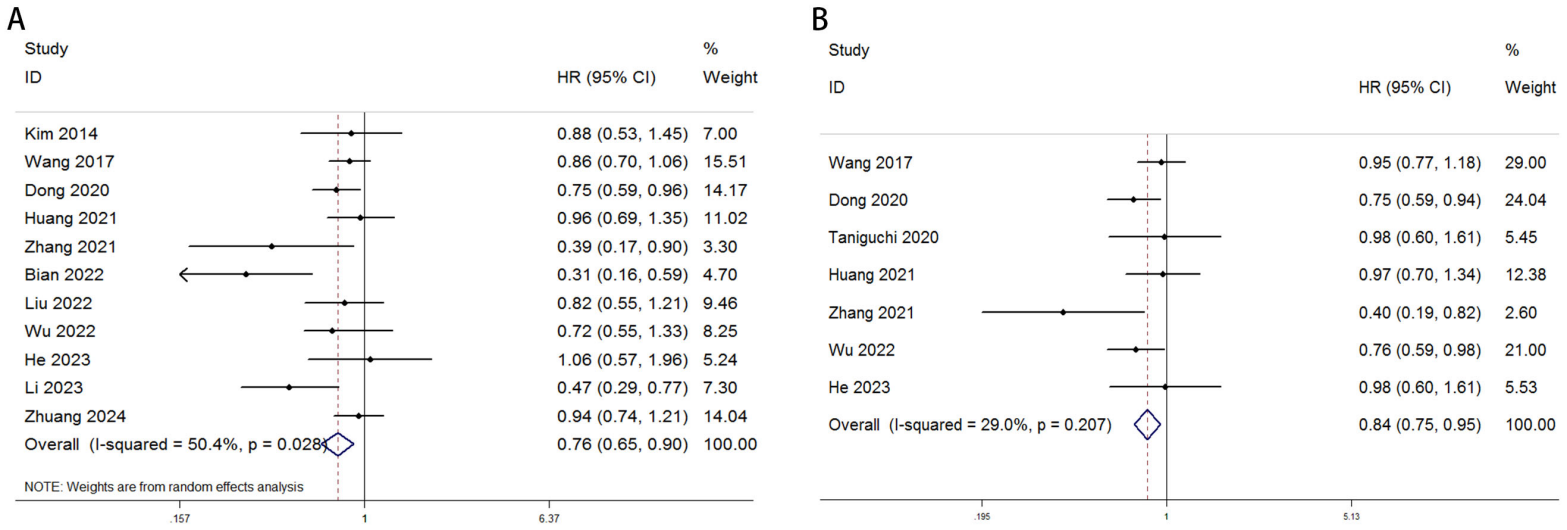
Forest plot illustrating the association between visceral fat area (VFA) and **(A)** overall survival (OS) and **(B)** disease-free survival (DFS) or recurrence-free survival (RFS) in gastric cancer patients.

A total of 11 studies, comprising 5,487 gastric cancer patients, investigated the association between VFA and OS. Heterogeneity analysis revealed statistically significant heterogeneity (P=0.028 < 0.1, I² = 50.4% > 50%), indicating that a random-effects model was the most appropriate for this meta-analysis. The pooled Hazard Ratio (HR) and corresponding 95% Confidence Interval (CI) were: HR=0.76, 95% CI=0.65–0.90, P=0.001, suggesting that elevated VFA correlates with improved OS in GC patients undergoing surgical or other therapeutic interventions (**Figure 2A**). Furthermore, seven studies, encompassing 4,023 patients, assessed the relationship between VFA and DFS/RFS in GC patients following surgery or other treatments. Heterogeneity testing indicated no significant heterogeneity (P=0.207 > 0.1, I² = 29.2% < 50%). The aggregated data demonstrated an HR of 0.84, 95% CI=0.75–0.95, P=0.004, indicating that higher VFA levels are associated with superior DFS or RFS outcomes in GC patients (**Figure 2B**). The prognostic value of VFA for OS in gastric cancer patients varied significantly across subgroups, with notable heterogeneity. Large-sample studies (>500 patients) demonstrated a significant association between lower VFA and improved OS (HR = 0.85, 95% CI: 0.76–0.96, P = 0.009), supported by low heterogeneity (I² = 0%), indicating robust results. In contrast, smaller studies (<500 patients) showed a lower HR (HR = 0.62, P = 0.01) but with higher heterogeneity (I² = 61.8%), necessitating cautious interpretation. When a cutoff of 100 cm² was applied, VFA significantly correlated with OS (HR = 0.86, P = 0.021) and minimal heterogeneity. However, studies using non-100 cutoffs suggested stronger protective effects (HR = 0.64, P = 0.004) but with moderate heterogeneity (I² = 54.7%), reflecting potential variability in cutoff selection. Prospective studies (HR = 0.77, P < 0.001) and multivariate-adjusted analyses (HR = 0.40, P < 0.001) further validated VFA as an independent prognostic factor. Retrospective studies, however, did not reach statistical significance (P = 0.058), possibly due to bias or confounding factors. Overall, lower VFA was consistently linked to prolonged OS, particularly in standardized, large-sample prospective cohorts. (**Tables 3**). The association between VFA and DFS/PFS was less consistent, with greater variability across subgroups. Large-sample studies (>500 patients) revealed a significant benefit of lower VFA for DFS (HR = 0.85, P < 0.001) and low heterogeneity (I² = 0%). However, smaller studies (<500 patients) showed a wide confidence interval (HR = 0.65, 95% CI: 0.27–1.57, P < 0.001) and extreme heterogeneity (I² = 75.7%), indicating unstable results. A cutoff of 100 cm² did not yield statistical significance for DFS (P = 0.07), while non-100 cutoffs showed a significant protective effect (HR = 0.75, P = 0.01) but with moderate heterogeneity (I² = 51.6%). Prospective studies supported a clear association (HR = 0.83, P = 0.005), whereas retrospective analyses were nonsignificant (P = 0.319). Notably, multivariate-adjusted results for DFS were inconclusive (HR = 0.65, P = 0.336), likely due to limited sample size (only 2 studies) or variability in adjusted confounders. These findings suggest that the prognostic role of VFA in DFS/PFS may depend on endpoint definitions, study design, and methodological heterogeneity. Future studies should prioritize standardized cutoffs, larger cohorts, and prospective designs to clarify its clinical utility for DFS/PFS (**Tables 4**).

**Table 3:** Subgroup analysis evaluating the prognostic significance of VFA for OS in gastric cancer patients undergoing surgery or other treatments.

Subgroup	NO. of studies	HR (95% CI)	P	Heterogeneity		Model
				I^2^ (%)	Ph	
Sample size						
>500	5	0.85 (0.76-0.96)	0.009	0	0.618	Fixed
<500	6	0.62 (0.43-0.89)	0.01	61.8	0.023	Random
Cuf-off						
100	4	0.86 (0.76-0.98)	0.021	0	0.564	Fixed
≠100	7	0.64 (0.47-0.86)	0.004	54.7	0.039	Random
Study design						
Retrospective	7	0.77 (0.6-1.01)	0.058	57.7	0.028	Random
Prospective	4	0.77 (0.67-0.89)	<0.001	42.1	0.159	Fixed
Analysis						
Univariate	8	0.86 (0.77-0.96)	0.006	0	0.869	Fixed
Multivariate	3	0.40 (0.28-0.57)	<0.001	0	0.589	Fixed

**Table 4:** Subgroup analysis evaluating the prognostic significance of VFA for DFS/RFS in gastric cancer patients undergoing surgery or other treatments.

Subgroup	NO. of studies	HR (95% CI)	P	Heterogeneity		Model
				I^2^ (%)	Ph	
Sample size						
>500	5	0.85 (0.76-0.96)	<0.001	0	0.421	Fixed
<500	2	0.65 (0.27-1.57)	<0.001	75.7	0.042	Random
Cuf-off						
100	4	0.88 (0.77-1.01)	0.07	0	0.408	Fixed
≠100	3	0.75 (0.61-0.93)	0.01	51.6	0.127	Fixed
Study design						
Retrospective	4	0.89 (0.71-1.12)	0.319	43.7	0.149	Fixed
Prospective	3	0.83 (0.2-0.94)	0.005	28.6	0.246	Fixed
Analysis						
Univariate	5	0.85 (0.76-0.96)	0.009	0	0.418	Fixed
Multivariate	2	0.65 (0.27-1.56)	0.336	75.4	0.044	Random

### 3.3 Publication bias and sensitivity analysis

**Figure 3:**
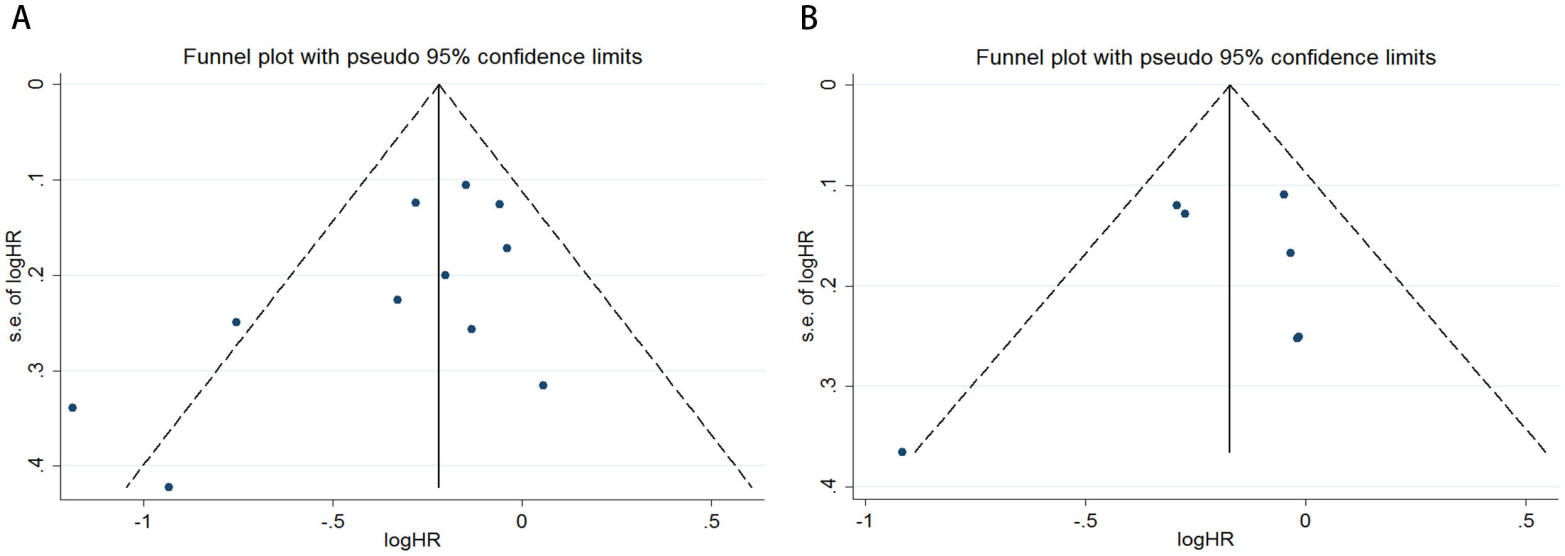
Funnel plots are utilized to assess the presence of publication bias in **(A)** overall survival (OS) and **(B)** disease-free survival (DFS) or recurrence-free survival (RFS).

**Figure 4:**
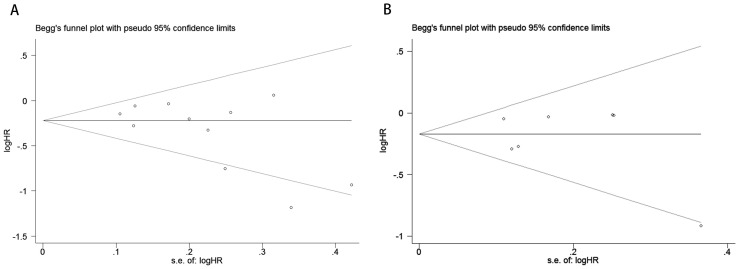
Publication bias test. **(A)** Begg tests for OS, p = 0.161; **(B)** Begg tests for DFS/RFS, p = 0.368.

**Figure 5:**
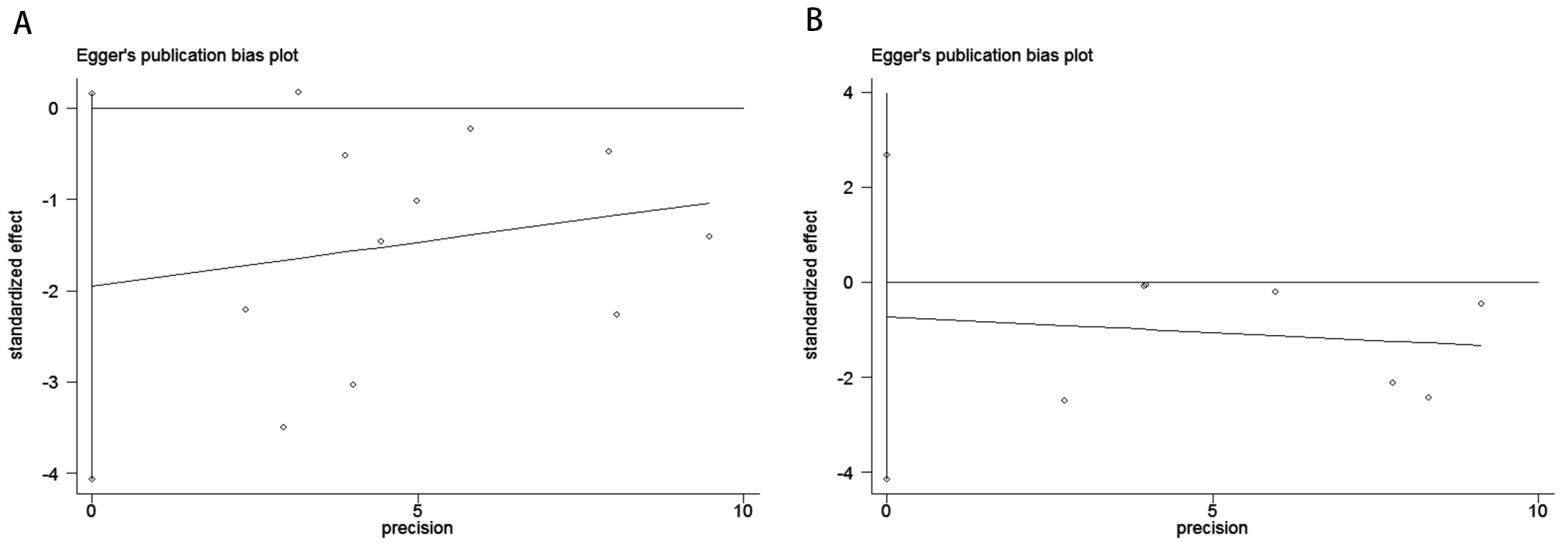
Publication bias test. **(A)** Egger’s test for OS, p = 0.067; **(B)** Egger’s test for DFS/RFS, p = 0.609.

**Figure 6:**
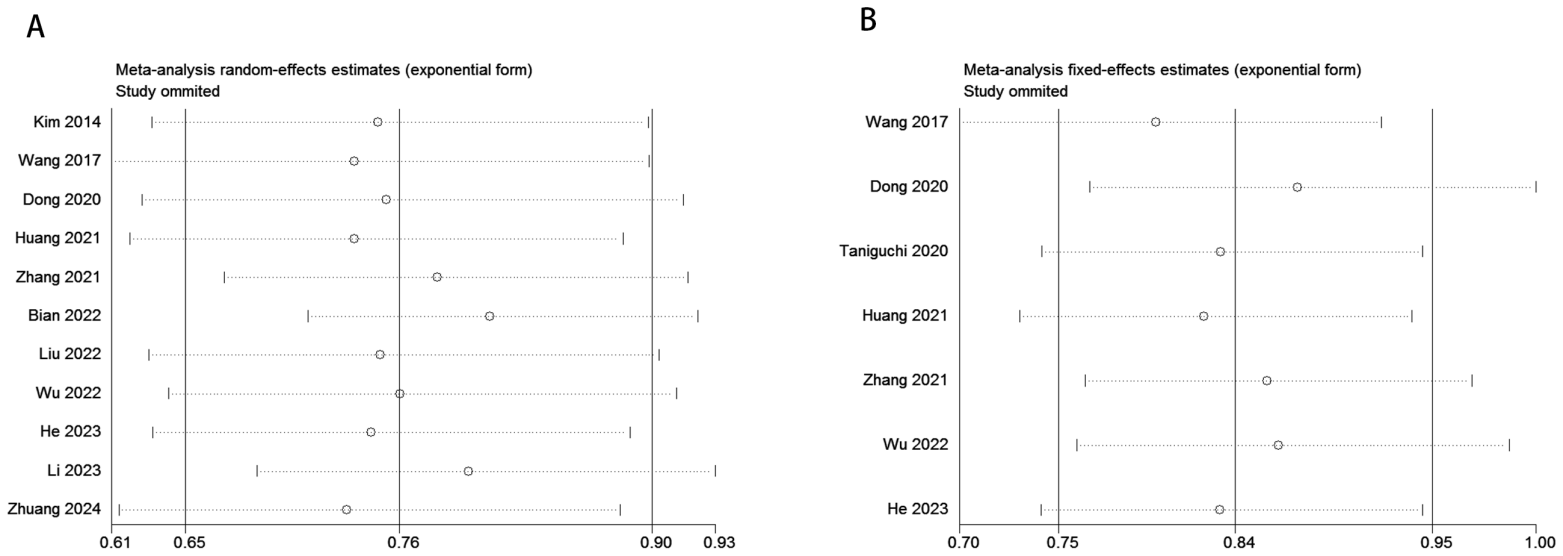
Sensitivity analysis for the pooled results between VFA and **(A)** OS and **(B)** DFS/RFS.

To evaluate potential publication bias, a combination of funnel plots, Begg’s test, and Egger’s test was utilized. The funnel plots for OS (**Figure 3A**) and DFS/RFS (**Figure 3B**) both displayed relatively symmetrical distributions. The results of Begg’s test indicated no significant publication bias for either OS or DFS/RFS (OS, p = 0.161; **Figure 4A**; DFS/RFS, p = 0.368; **Figure 4B**). Egger’s test further corroborated these findings, showing no evident publication bias in the studies related to OS and DFS/RFS (OS, p = 0.067; **Figure 5A**; DFS/RFS, p = 0.609; **Figure 5B**). To further assess the possibility of publication bias, we conducted a sensitivity analysis by sequentially excluding each study and performing a cumulative analysis to determine its impact on the overall results. The analysis demonstrated that no individual study had a significant effect on the relationship between VFA and OS or DFS/RFS in gastric cancer patients (**Figure 6**). This finding underscores the robustness of the observed associations, as the results remained consistent and unaffected by the exclusion of individual studies.

## 4 Discussion

Gastric cancer (GC) is a leading cause of cancer-related mortality worldwide. Despite significant advances in treatment over recent years, the recurrence and mortality rates for gastric cancer remain high (45, 46). Complications following surgical treatment, along with adverse events from chemotherapy, immunotherapy, and targeted therapy, severely impact patient survival outcomes. Multiple studies have indicated that excessive abdominal visceral fat not only affects metabolic function but is also closely associated with postoperative complications and adverse events in gastric cancer patients (38–41). Additionally, adipose tissue secretes various factors, such as tumor necrosis factor (TNF) and interleukin-6 (IL-6) (47, 48). These factors are not only associated with the mechanical effects of obesity, such as gastroesophageal reflux, but are also considered to play a significant pathological role in the development and progression of gastrointestinal cancers.Therefore, accurate prognostic assessment is crucial before performing radical gastric surgery or initiating non-surgical treatments such as combination chemotherapy.

BMI (Body Mass Index) is a commonly used indicator for assessing obesity, but it has significant limitations, particularly regarding accuracy across different ethnic groups. For instance, Asians often face a higher risk of cardiovascular diseases or metabolic syndrome at lower BMI levels (49). This discrepancy is largely due to differences in body fat distribution and percentage. BMI reflects only the ratio of weight to height, failing to distinguish between muscle and fat composition, and it does not effectively differentiate between peripheral obesity (fat accumulation in the limbs) and abdominal obesity (visceral fat accumulation), the latter of which is associated with greater health risks. Consequently, relying solely on BMI to assess health can overlook potential metabolic risks and variations in body composition. In contrast, visceral fat is a more reliable indicator of disease risk, with CT assessment considered the gold standard for detecting visceral fat obesity. Since Asians tend to accumulate visceral fat more readily, using visceral fat area (VFA) as an obesity assessment standard is more accurate than BMI and better reflects health risks.

Previous studies have shown that patients with higher intra-abdominal fat, such as those with pancreatic or colorectal cancer, tend to have poorer overall survival rates. This may be due to visceral fat increasing serum levels of inflammatory cytokines, angiogenic factors, and oxidative stress markers, thereby affecting the tumor microenvironment (50–52). However, in contrast to these findings, Harada et al. (53) demonstrated that low visceral fat could significantly increase overall mortality in patients with upper gastrointestinal cancers. Many researchers believe that high BMI or high VFA correlates with poor prognosis in gastric cancer patients, as previous studies have shown a strong association between high BMI or VFA and decreased postoperative morbidity and lymph node retrieval (54–56). Feng et al. (57) found that preoperative low VFA and significant postoperative VFA loss predicted shorter progression-free survival and overall survival in metastatic gastric cancer patients. Similarly, Park et al. (58) reported that a marked reduction in postoperative VFA was indicative of shorter progression-free and overall survival. Furthermore, Matsui et al. (59) identified low VFA as an independent prognostic factor for poor adherence to adjuvant chemotherapy in advanced gastric cancer patients requiring neoadjuvant treatment. Likewise, Zhang et al. (36) noted that lower VFA before and after neoadjuvant chemotherapy correlated with worse progression-free and overall survival. In summary, the impact of high VFA on survival outcomes in gastric cancer patients post-surgery or after non-surgical treatment remains unclear.

The purpose of this study was to determine whether VFA can serve as a prognostic indicator for assessing the prognosis of gastric cancer patients after treatment. To clarify the impact of high VFA on survival outcomes in gastric cancer patients following surgery or non-surgical treatment, we conducted a comprehensive meta-analysis of data from 12 relevant trials involving 6,054 patients from three countries. Our analysis confirmed the favorable impact of higher visceral fat on the prognosis of gastric cancer patients. Patients with higher visceral fat had longer overall survival (OS), disease-free survival (DFS), and recurrence-free survival (RFS) compared to those with lower visceral fat. The pooled hazard ratio (HR) for OS was 0.76 (95% CI = 0.65–0.90, P = 0.001), and the pooled HR for DFS or RFS was 0.84 (95% CI = 0.75–0.95, P = 0.004). This suggests that the beneficial impact of visceral fat on the prognosis of gastric cancer patients outweighs its potential harmful effects. In addition, our subgroup analysis indicated that higher VFA was predictive of more favorable OS and DFS or RFS outcomes regardless of sample size (≥500 or <500), cut-off value (100 or ≠100), study design (retrospective or prospective), and analysis type (multivariate or univariate). Notably, heterogeneity in OS might be attributed to differences in studies with sample sizes of <500, cut-off values ≠100, or retrospective designs. Heterogeneity in DFS or RFS might be due to differences in sample sizes <500 or studies using multivariate analysis. To assess potential publication bias, we employed several methods, including funnel plot analysis, Begg’s test, and Egger’s test. Sensitivity analysis and assessment of publication bias further corroborated the robustness of the conclusions drawn in this meta-analysis. Some clinical trials found no significant correlation between VFA and prognosis in gastric cancer patients. We consider the following possible reasons for this inconsistency: First, insufficient sample sizes in some trials may have led to result bias (35). Second, differences in baseline characteristics among patients could lead to variations in treatment outcomes. For example, in Taniguchi’s study, a higher proportion of patients with low visceral fat content received neoadjuvant or adjuvant therapy, which improved the long-term prognosis of this group (40). Third, treatment protocols varied across different centers. When the negative impact of higher VFA outweighs its benefits, the correlation with favorable prognosis may be obscured. There are several limitations to our study. First, there was heterogeneity among the included studies. Patient baseline characteristics, VFA cut-off values, and treatment protocols varied across trials. Second, the sample size in this analysis was relatively limited, with most data derived from Asian countries. Therefore, the value of VFA in European and other populations requires further exploration to determine its applicability across different demographics. Third, despite differences in body composition and hormones between men and women, we were unable to conduct a gender-based analysis due to the limitations of the original literature. Consequently, more high-quality, large-sample, prospective studies are needed in the future to validate and refine our findings.

## 5 Conclusions

This meta-analysis demonstrated that among patients with gastric cancer who underwent surgery or other treatments, elevated VFA was significantly associated with improved OS and DFS. Consequently, VFA may serve as a useful prognostic indicator for assessing the prognosis of gastric cancer patients following treatment. However, further prospective studies are necessary to validate these findings and confirm the reliability of VFA as a prognostic marker.

## Data Availability

The original contributions presented in the study are included in the article/supplementary material. Further inquiries can be directed to the corresponding author.
